# Supplementary material for: Gait Training in Virtual Reality: Short-Term Effects of Different Virtual Manipulation Techniques in Parkinson’s Disease
Source: Cells. 2019 May 6;8(5):419. doi: 10.3390/cells8050419 (PMC6562780; doi:10.3390/cells8050419)
Supplement: Supplementary file 1 [file cells-08-00419-s001.pdf]

Article – Supplementary Files

Gait Training in Virtual Reality: Short-Term Effects of Different Virtual Manipulation Techniques in Parkinson's Disease

Omar Janeh[1,#], Odette Fründt[2,#], Beate Schönwald[2], Alessandro Gulberti[2,3], Carsten Buhmann[2], Christian Gerloff[2], Frank Steinicke[1,‡], Monika Pötter-Nerger[2,‡*]

[1] University of Hamburg, Department of Informatics, Human Computer Interaction, Hamburg, Germany, Emails: omar.janeh@gmail.com (O.J.), frank.steinicke@uni-hamburg.de (F.S.)

[2] University Medical Center Hamburg-Eppendorf, Department of Neurology, Hamburg, Germany, Emails: o.fruendt@uke.de (O.F.), b.schoenwald@uke.de (B.S.), buhmann@uke.de (C.B.), gerloff@uke.de (C.G.), m.poetter-nerger@uke.de (M.P.)

[3] University Medical Center Hamburg-Eppendorf, Department of Neurophysiology and Pathophysiology, Hamburg, Germany, Email: agulberti@uke.de (A.G.)

* Correspondence: m.poetter-nerger@uke.de; Tel.: +0049-152/228 27 457, Fax: +0049-40/40093 (M.P.)

Supplementary Files

Supplementary Information on gait velocity during each of the three trials during each condition

We additionally performed an overall ANOVA including each of the three trials (factor trial) and all the conditions (factor condition). For all conditions, during the first trial gait velocity was significantly slower ($p < 0.05$) compared to trials 2 and 3 whereas gait velocity during the trials 2 and 3 did not differ significantly.

Mean trial 1: 98.68, SD: 15.95
Mean trial 2: 102.51, SD: 15.19
Mean trial 3: 103.62, SD: 15.01

Trial effects: $F_{1.63, 20.89}$ = 37,137, p = 0.001, $\eta_p^2 = 0.726$.
Condition effects: $F_{4.36, 61.15}$ = 5,982, p = 0.001, $\eta_p^2 = 0.299$.
Trail x Condition : $F_{5.43, 76.12}$ = 1,683, p = 0.143, $\eta_p^2 = 0.107$.

Supplementary Figures S1 and S2:

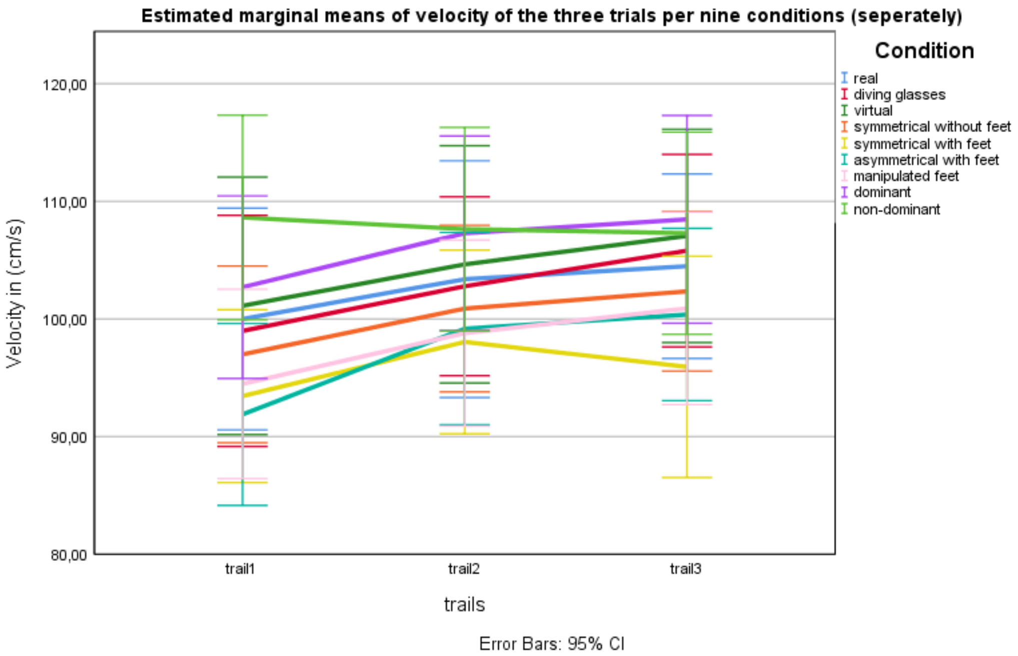

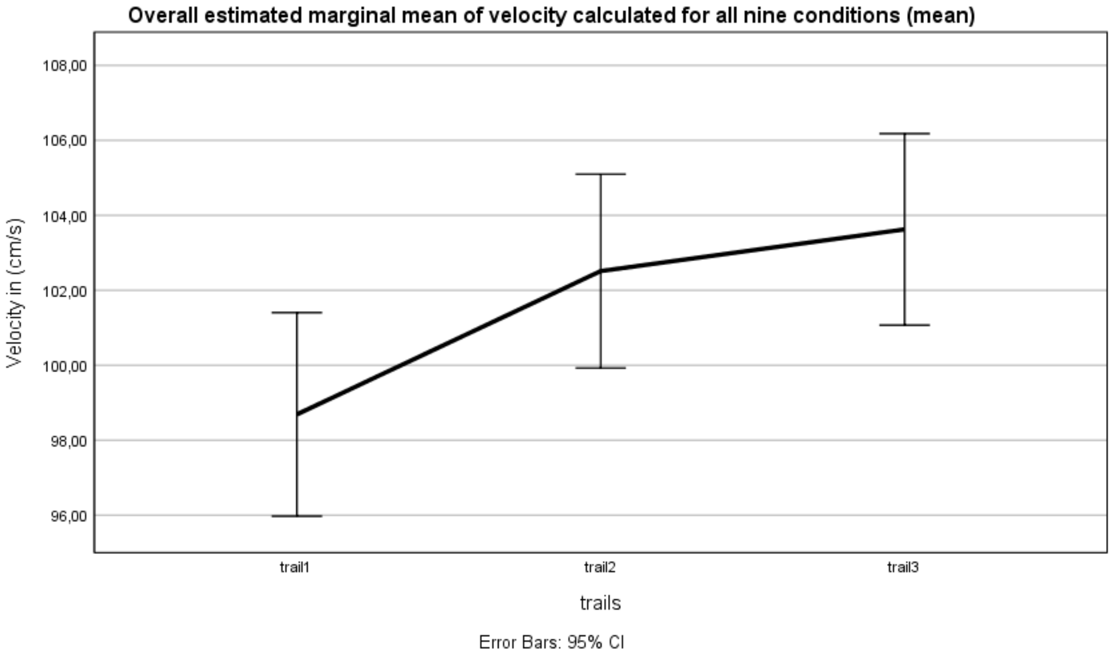

Figures S1 and S2. Results of the overall ANOVA for gait velocity including each of the three trials (factor trial) and all the conditions (factor condition). Figure S1 shows the mean gait velocity for trials 1,2 and 3 for each condition that are marked with different colors. Figure S2 shows the mean gait velocity of all conditions during trials 1,2 and 3. In all conditions, during the first trial gait velocity was significantly slower ($p < 0.05$) compared to trials 2 and 3 whereas gait velocity during the trials 2 and 3 did not significantly differ.

Table S1: Spatiotemporal parameters of gait, gait variability CV* (%) and gait asymmetry measured during “non-MLS phase” (conditions without motor learning strategies) of the experiment.

**Conditions Without Specific Motor Learning Strategies (non-MLS)**			
**Parameter**	**Mean +/- SD**	**Mean +/- SD**	**Mean +/- SD**

		Baseline	Body side Comparison	Diving glasses	Body side Comparison	Real virtual	Body side Comparison
Step length (cm)	S	**58.34 ± 8.27**	##	**57.60 ± 7.77**	##	**57.18 ± 9.45**	##
	L	**61.34 ± 7.78**		**61.33 ± 7.91**		**60.94 ± 9.26**	
Velocity (cm/s)		102.61 ± 16.22		102.52 ± 14.59		104.26 ± 17.82	
Cadence (step/min)		102.81 ± 8.19		103.48 ± 6.67		**106.42 ± 8.42****	
Gait asymmetry		1.05 ± 0.04		1.06 ± 0.04		**1.07 ± 0.05***	
Stride Velocity (cm/s)	S	104.19 ± 16.54		104.22 ± 14.68		105.98 ± 17.99	
	L	104.42 ± 16.89		104.70 ± 15.32		106.73 ± 19.02	
Step time (s)	S	**0.5827 ± 0.04951**	#	0.5789 ± 0.04372		**0.5616 ± 0.04786** **	##
	L	**0.5921 ± 0.04409**		0.5855 ± 0.03575		**0.5767 ± 0.04370** **	
Step width (cm)	S	10.06 ± 3.55		10.30 ± 3.47		**11.66 ± 3.96****	
	L	10.41 ± 3.54		10.61 ± 3.62		**11.74 ± 3.85****	
Double support time (s)	S	0.3372 ± 0.06058		0.3336 ± 0.04784		0.3311 ± 0.05515	
	L	0.3374 ± 0.05748		0.3347 ± 0.04733		0.3323 ± 0.05506	
Swing time (s)	S	0.4119 ± 0.03417		0.4076 ± 0.03222		**0.3919 ± 0.03970****	
	L	0.4085 ± 0.03475		0.4030 ± 0.03211		**0.3923 ± 0.03973****	
Step length CV (%)	S	4.53 ± 2.31		5.45 ± 2.56		5.63 ± 2.42	
	L	4.64 ± 2.01		4.24 ± 1.72		5.69 ± 2.61	
Stride Velocity CV (%)	S	5.58 ± 2.25		5.76 ± 2.22		6.95 ± 2.83	
	L	5.14 ± 1.98		5.38 ± 2.43		6.22 ± 2.66	

Step time CV (%)	S	5.23 ± 2.27		5.43 ± 2.52		**5.62 ± 2.95***	
	L	5.67 ± 2.31		5.91 ± 2.61		**6.97 ± 2.46***	
Step width CV (%)	S	22.57 ± 12.57		23.01 ± 13.99		21.36 ± 15.17	
	L	22.00 ± 14.01		24.23 ± 15.15		21.73 ± 14.89	
Double support time CV (%)	S	6.24 ± 2.13		6.31 ± 1.83		6.94 ± 2.28	
	L	6.92 ± 2.25		5.92 ± 2.13		7.20 ± 2.39	
Swing time CV (%)	S	**4.25 ± 1.45**	##	**4.31 ± 1.28**	##	**5.22 ± 2.31**	##
	L	**3.81 ± 1.31**		**3.98 ± 1.28**		**4.00 ± 1.89**	
FAP score		89.26 ± 13.47		88.53 ± 12.57		86.4 ± 14.27	

This table gives an overview about the spatiotemporal gait parameters including gait variability CV* (%) and gait asymmetry measured during the "non-MLS conditions" of the experiment.
Abbreviations: Significantly different to the baseline: (*) = $p < 0.05$, (**) = $p < 0.01$. Significant difference between short and long side: (#) = $p < 0.05$, (##) = $p < 0.01$. S = leg with shorter step length, L = leg with longer step length. CV = coefficient of variation.

Table S2. Spatiotemporal parameters of gait, gait variability CV* (%) and gait asymmetry measured during the "MLS phase" (conditions with specific motor learning strategies) of the experiment.

**Conditions with Motor Learning Strategies (MLS)**									
		Mean +/- SD							
Parameter		Baseline	Body side Comparison	Symmetrical without feet	Symmetrical with feet	Body side Comparison	Asymmetrical with feet	Body side Comparison	Manipulated foot
	S	58.34 ± 8.27	##	59.87 ± 7.91	59.21 ± 8.11	#	61.10 ± 7.84a**	#	60.45 ± 8.16

Step length (cm)	L	61.34 ± 7.78		61.17 ± 7.17	61.45 ± 6.97		62.96 ± 7.36a**		60.80 ± 7.68
Velocity (cm/s)		102.61 ± 16.22		100.06 ± 12.49	95.8 ± 13.88		97.14 ± 13.48		98.05 ± 14.08
Cadence (step/min)		102.81 ± 8.19		99.66 ± 9.65	95.32 ± 7.69**		93.98 ± 7.28**		97.41 ± 9.9
Gait asymmetry (%)		1.05 ± 0.04		1.02 ± 0.05	1.04 ± 0.07		1.03 ± 0.05		1.01 ± 0.06*
Stride Velocity (cm/s)	S	104.19 ± 16.54		101.40 ± 12.41	97.08 ± 13.82		98.46 ± 13.59		99.69 ± 13.99
	L	104.42 ± 16.89		101.99 ± 13.53	97.73 ± 14.79		99.08 ± 14.59		100.06 ± 15.47
Step time (s)	S	0.5827 ± 0.04951		0.6035 ± 0.06951	0.6289 ± 0.06556*		0.6356 ± 0.06050 **		0.6224 ± 0.09013
	L	0.5921 ± 0.04409		0.6128 ± 0.07552	0.6404 ± 0.04854*		0.6498 ± 0.05524 **		0.6245 ± 0.07853
Step width (cm)	S	10.06 ± 3.55		11.91 ± 3.26**	12.71 ± 3.65**		12.84 ± 3.98**		12.98 ± 4.01**
	L	10.41 ± 3.54		11.90 ± 3.22**	12.83 ± 3.62**		12.74 ± 4.03**		13.05 ± 4.02**
Double support time (s)	S	0.3372 ± 0.06058		0.3335 ± 0.05559	0.3525 ± 0.05499		0.3528 ± 0.05730		0.3413 ± 0.05793
	L	0.3374 ± 0.05748		0.3346 ± 0.05284	0.3522 ± 0.05094		0.3528 ± 0.05300		0.3404 ± 0.05473
Swing time (s)	S	0.4119 ± 0.03417		0.4268 ± 0.05315	0.4482 ± 0.04569*		0.4521 ± 0.04504 **		0.4394 ± 0.07250

	L	0.4085 ± 0.03475		0.4261 ± 0.07230	0.4441 ± 0.04818*		0.4535 ± 0.06333 **		0.4375 ± 0.08369
Stride Velocity CV (%)	S	5.58 ± 2.25		5.24 ± 2.90	5.40 ± 2.60		5.14 ± 2.74		5.72 ± 3.11
	L	5.14 ± 1.98		4.98 ± 2.56	5.08 ± 2.83		4.38 ± 1.94		5.04 ± 2.33
Step length CV (%)	S	4.53 ± 2.31		4.57 ± 1.26	4.95 ± 1.56		4.96 ± 1.50		4.58 ± 1.41
	L	4.64 ± 2.01		4.48 ± 1.65	5.32 ± 2.93		3.74 ± 1.02		4.71 ± 1.53
Step time CV (%)	S	5.23 ± 2.27		7.59 ± 3.58**	7.29 ± 3.48**		6.16 ± 2.86		7.69 ± 3.69**
	L	5.67 ± 2.31		9.30 ± 3.78**	9.75 ± 4.40**		7.96 ± 3.32		9.07 ± 3.82**
Step width CV (%)	S	22.57 ± 12.57		19.59 ± 9.07	20.05 ± 10.00		19.11 ± 10.71		15.44 ± 5.01
	L	22.00 ± 14.01		17.61 ± 6.75	19.60 ± 12.70		20.94 ± 12.98		17.13 ± 6.27
Double support time CV (%)	S	6.24 ± 2.13		6.46 ± 1.48	6.22 ± 2.51		6.45 ± 2.73		6.46 ± 2.08
	L	6.92 ± 2.25		6.82 ± 1.93	6.35 ± 2.79		6.47 ± 2.64		6.52 ± 2.12
Swing time CV (%)	S	4.25 ± 1.45		5.99 ± 2.60*	7.92 ± 4.44*		5.59 ± 1.89*		6.46 ± 2.12*
	L	3.81 ± 1.31		6.56 ± 3.05*	7.34 ± 5.14*		5.18 ± 2.11*		6.48 ± 2.21*
FAP score		89.26 ± 13.47		87.8 ± 12.8	86.6 ± 13.08		87.75 ± 12.51		86.2 ± 14.32

This table gives an overview about the spatiotemporal gait parameters including gait variability CV* (%) and gait asymmetry measured during the “MLS conditions” (conditions with specific motor learning strategies) of the experiment.

Abbreviations: (*) = $p < 0.05$, (**) = $p < 0.01$ and (***) = $p < 0.001$ = Significantly different to the baseline.
(#) = $p < 0.05$, (##) = $p < 0.01$ and (###) = $p < 0.001$ = Significant difference between short and long side.
S = leg with shorter step length, L = leg with longer step length. CV = coefficient of variation.

Table S3. Spatiotemporal parameters of gait, gait variability CV* (%) and gait asymmetry measured during the testing for after-effects of the experiment.

**Testing for After-Effects**							
		Baseline	Body side Comparison	Dominant	Body side Comparison	Non-dominant	Body side Comparison
Step length (cm)	S	58.34 ± 8.27	##	60.27 ± 8.11	##	61.04 ± 8.34	##
	L	61.34 ± 7.78		62.99 ± 8.55		63.90 ± 7.96	
Velocity (cm/s)		102.61 ± 16.22		106.14 ± 14.61		107.84 ± 15.26	
Cadence (step/min)		102.81 ± 8.19		103.57 ± 7.12		103.79 ± 7.92	
Gait asymmetry (%)		1.05 ± 0.04		1.04 ± 0.04		1.05 ± 0.05	
Stride Velocity (cm/s)	S	104.19 ± 16.54		107.78 ± 14.62		110.03 ± 15.64	
	L	104.42 ± 16.89		108.26 ± 15.36		109.63 ± 15.41	
Step time (s)	S	0.5827 ± 0.04951		0.5805 ± 0.04649		0.5774 ± 0.04304	
	L	0.5921 ± 0.04409		0.5832 ± 0.03544		0.5842 ± 0.04877	

Step width (cm)	S	10.06 ± 3.55		10.53 ± 3.73		10.69 ± 3.51	
	L	10.41 ± 3.54		10.79 ± 3.57		10.77 ± 3.73	
Double support time (s)	S	0.3372 ± 0.06058		0.3287 ± 0.05239		0.3218 ± 0.05019	
	L	0.3374 ± 0.05748		0.3296 ± 0.05257		0.3222 ± 0.04944	
Swing time (s)	S	0.4119 ± 0.03417		0.4105 ± 0.03295		0.4082 ± 0.03367	
	L	0.4085 ± 0.03475		0.4028 ± 0.03677		0.4094 ± 0.04003	
Stride Velocity CV (%)	S	5.58 ± 2.25		5.31 ± 2.12		5.60 ± 2.18	
	L	5.14 ± 1.98		5.18 ± 2.20		5.23 ± 2.50	
Step length CV (%)	S	4.53 ± 2.31		5 ± 2.5		4.82 ± 2.08	
	L	4.64 ± 2.01		4.75 ± 2.7		4.44 ± 1.75	
Step time CV (%)	S	5.23 ± 2.27		5.58 ± 2.64		6.55 ± 3.13	
	L	5.67 ± 2.31		6.35 ± 2.87		5.57 ± 3	
Step width CV (%)	S	22.57 ± 12.57		24.15 ± 16.76		18.44 ± 11.52	
	L	22.00 ± 14.01		23.53 ± 17.63		21.11 ± 11.80	
Double support time CV (%)	S	6.24 ± 2.13		6.11 ± 2.19		6.38 ± 2.50	
	L	6.92 ± 2.25		6.45 ± 1.78		6.62 ± 2.45	
Swing time CV (%)	S	4.25 ± 1.45		4.23 ± 2.13		4.40 ± 1.80	
	L	3.81 ± 1.31		3.94 ± 1.62		3.97 ± 1.43	

FAP score		89.26 ± 13.47		90.2 ± 13.04		90.48 ± 13.49	

This table gives an overview about the spatiotemporal gait parameters including gait variability CV* (%) and gait asymmetry measured during the testing for after-effects of the experiment.

Abbreviations: (*) = $p < 0.05$, (**) = $p < 0.01$ and (***) = $p < 0.001$ = Significantly different to the baseline

(#) = $p < 0.05$, (##) = $p < 0.01$ and (###) = $p < 0.001$ = Significant difference between short and long side

S = leg with shorter step length, L = leg with longer step length

CV = coefficient of variation
